# Scale up of the learning circles: a participatory action approach to support local food systems in four diverse First Nations school communities within Canada

**DOI:** 10.1186/s12889-024-19391-z

**Published:** 2024-08-15

**Authors:** Ashleigh Domingo, Jennifer Yessis, Barbara Zupko, Louise Watson McEachern, Renata Valaitis, Kelly Skinner, Rhona M. Hanning

**Affiliations:** 1 School of Public Health Sciences, University of Waterloo, Waterloo, ON N2L 3G1 Canada; 2 Department of Human Health and Nutritional Sciences, University of Guelph, Guelph, ON N1G 2W1 Canada; 3 Department of Geography, Western University, London, ON N6A 3K7 Canada

**Keywords:** First Nations, Community health promotion, Participatory research, Implementation science, Learning circle, Collaboration, Food systems, Food security, Food sovereignty, Scale-up

## Abstract

**Background:**

Addressing Indigenous food security and food sovereignty calls for community-driven strategies to improve access to and availability of traditional and local food. Participatory approaches that integrate Indigenous leadership have supported successful program implementation. Learning Circles: Local Healthy Food to School is a participatory program that convenes a range of stakeholders including food producers, educators and Knowledge Keepers to plan, implement and monitor local food system action. Pilot work (2014–2015) in Haida Gwaii, British Columbia (BC), showed promising results of the Learning Circles (LC) approach in enhancing local and traditional food access, knowledge and skills among youth and adolescents. The objective of the current evaluation was therefore to examine the process of scaling-up the LC vertically within the Haida Nation; and horizontally across three diverse First Nations contexts: Gitxsan Nation, Hazelton /Upper Skeena, BC; Ministikwan Lake Cree Nation, Saskatchewan; and Black River First Nation, Manitoba between 2016 and 2019.

**Methods:**

An implementation science framework, Foster-Fishman and Watson’s (2012) ABLe Change Framework, was used to understand the LC as a participatory approach to facilitate community capacity building to strengthen local food systems. Interviews (*n* = 52), meeting summaries (*n* = 44) and tracking sheets (*n* = 39) were thematically analyzed.

**Results:**

The LC facilitated a collaborative process to: (1) build on strengths and explore ways to increase readiness and capacity to reclaim traditional and local food systems; (2) strengthen connections to land, traditional knowledge and ways of life; (3) foster community-level action and multi-sector partnerships; (4) drive actions towards decolonization through revitalization of traditional foods; (5) improve availability of and appreciation for local healthy and traditional foods in school communities; and (6) promote holistic wellness through steps towards food sovereignty and food security. Scale-up within Haida Gwaii supported a growing, robust local and traditional food system and enhanced Haida leadership. The approach worked well in other First Nations contexts, though baseline capacity and the presence of champions were enabling factors.

**Conclusions:**

Findings highlight LC as a participatory approach to build capacity and support iterative planning-to-action in community food systems. Identified strengths and challenges support opportunities to expand, adopt and modify the LC approach in other Indigenous communities with diverse food systems.

## Background

Protecting traditional food practices and relationships to the land against ongoing colonial pressures has long been of importance to maintaining Indigenous well-being in communities across Canada. Community programs have increasingly focused on ways to revitalize local food systems as a pathway to greater food security taking steps towards food sovereignty [1, 2]. Successful implementation of such endeavors has valued community engagement and centred Indigenous perspectives and strengths in program planning [1–9]. Non-Indigenous partners in program planning have looked to strong Indigenous leadership and community collaboration to ensure actions are community-driven and reflective of community priorities. This greater movement towards fostering strong relationships and co-development has been supported by participatory approaches, which emphasize collaboration and co-production of knowledge to advance social change and health equity [10–13].

The Learning Circles: Local Healthy Food to School (LC: LHF2S) is a participatory program that was developed in collaboration with communities. The goals of the initiative were to strengthen community capacity to enhance local and traditional healthy food access, knowledge and skills among school communities. In Haida Gwaii, the traditional home of the Haida People, ‘Learning Circles’ (LC) model, as it became known, was adapted from the US Farm to School ‘Learning Labs’ by Farm to Cafeteria Canada in 2014 [14]. This was the first time the model had been applied within an Indigenous context in Canada. The LC as an approach to strengthening food-health related community priorities, supports a participatory community engagement practice. The LC fosters multi-sector relationships, collaboration, and shared decision-making with a broad range of people with interest in locally grown and, as emerged, traditional food [15–21]. The process is supported by a LC facilitator (LCF) who brings a range of community members together such as hunters, gatherers, farmers, food producers, Knowledge Keepers, school staff, families and students to engage in an iterative process of planning, prioritizing and re-assessing local food actions.

Scale-up has been described as efforts to increase the impact of a successfully evaluated intervention to benefit more people and to foster maintenance and sustainability [22]. Horizontal scale-up includes expansion or replication of a project or intervention; while vertical scale-up refers to focused efforts on the changes needed to support institutionalization [22]. While piloted work of the LC showed promising results (2014–2015), it was unknown at the start of the current evaluation in 2016 how the LC approach to building capacity within local school community food systems would support ongoing work in Haida Gwaii (vertical scale-up) and apply in diverse First Nations communities (horizontal scale-up). We therefore explored whether an approach that had been successful in one Indigenous context could be applied in different contexts. Enabling features of successful implementation were examined in each community.

Little is known about scaling-up initiatives in Indigenous contexts with diverse traditions, governance structures and relationships that influence readiness and capacity for community food actions [23, 24]. Few published studies have evaluated implementation of community-identified food-related projects within Indigenous communities. Several small-scale studies highlight the importance of enabling factors: project champions to oversee planning and implementation, a shared sense of community ownership, time and commitment, access to resources, and knowledge of food sustainability practices [2, 9]. Others highlight the importance of understanding the social and cultural context in which interventions are implemented to foster reciprocal relationships between the program and its context [5, 25, 26], co-governance mechanisms to ensure transparency and accountability in decision making [25], community collaboration and partnerships [27], and integration of a dynamic and process-oriented approach that can guide and foster community participation in planning, implementation and evaluation [5].

In this paper we use an implementation science framework, Foster-Fishman and Watson’s (2012) ABLe Change Framework [28], to understand the process of scaling-up the LC model across four diverse contexts. Hence, the objective was to examine features of the implementation of the LC process within four partnering First Nations communities from 2016 to 2019: Haida Nation, Haida Gwaii, British Columbia (BC); Gitxsan Nation, Hazelton /Upper Skeena, BC; Ministikwan Lake Cree Nation, Saskatchewan (SK); and Black River First Nation, Manitoba (MB). Findings from this work highlight the LC as a participatory approach to build capacity and support iterative planning-to-action within local food systems. Strengths and challenges discussed will support opportunities to expand, adopt and modify the LC approach in other Indigenous communities with diverse food systems.

## Methods

### LHF2S: project advisory structure

Partnerships within the LHF2S initiative were governed by two advisory groups: Local LC Council and Project Stakeholder Advisory Council. Within each community, a local LC council was established with community members representing local governance and key community partners encompassing leadership within health, schools or partnering organizations. The LC council oversaw research processes, including hiring and supporting of a LCF. The LCF, often a community member or ally with strong relationships established and connections to local food systems and school(s), moderated/supported and facilitated project planning and evaluation activities. In addition, the LCF engaged community members, convened partners at LC meetings and workshops, and provided ongoing communications and support for project activities prioritized by the LC. Coordination for the full cross-community project involved a Project Stakeholder Advisory Council consisting of the LCF and a representative from each community, core research team members and representatives of key partnering national health organizations, such as the Heart and Stroke Foundation. This group coordinated project progress, supported communications between communities, oversaw evaluation and knowledge mobilization activities and planned annual project gatherings.

In support of community leadership, funding and sharing agreements were administered by each community in partnership with the University of Waterloo. Ethics approval to pursue evaluation activities was obtained from the University of Waterloo Office of Research Ethics (ORE# 30819). The First Nations principles of Ownership, Control, Access and Possession (OCAP^®^) [29] were applied in the conduct of evaluation activities in addition to specific protocols taken as identified by partnering communities. The research was funded through a Canadian Institutes of Health Research initiative: Pathways to Health Equity for Indigenous Peoples, that aimed to further understandings of how to design and implement sustainable programs to improve Indigenous health and health equity [30]. Additional support was provided by Heart & Stroke Foundation of Canada and Farm to Cafeteria Canada.

### Project conceptualization and implementation initiation

Prior to the three-year period (2016–2019) during which the LC model was scaled up in four diverse communities, the Project Stakeholder Advisory Council shaped the initiative and secured funding. The opportunity to extend and evaluate the LC model began with an Expression of Interest to CIHR-Pathways (February 2014) and a seed grant through the Waterloo Chronic Disease Prevention Initiative (August 2014). This seed funding brought together partnering organizations, community contacts and research team members who had worked together on previous Indigenous food-health projects. Four communities became engaged in a subsequent proposal planning meeting in Haida Gwaii in 2015. Other organizations who became involved at this stage were the Native Women’s Association of Canada (a CIHR Pathways-identified Partner in Engagement and Knowledge Exchange) and Storytellers’ Foundation.

**Fig. 1:**
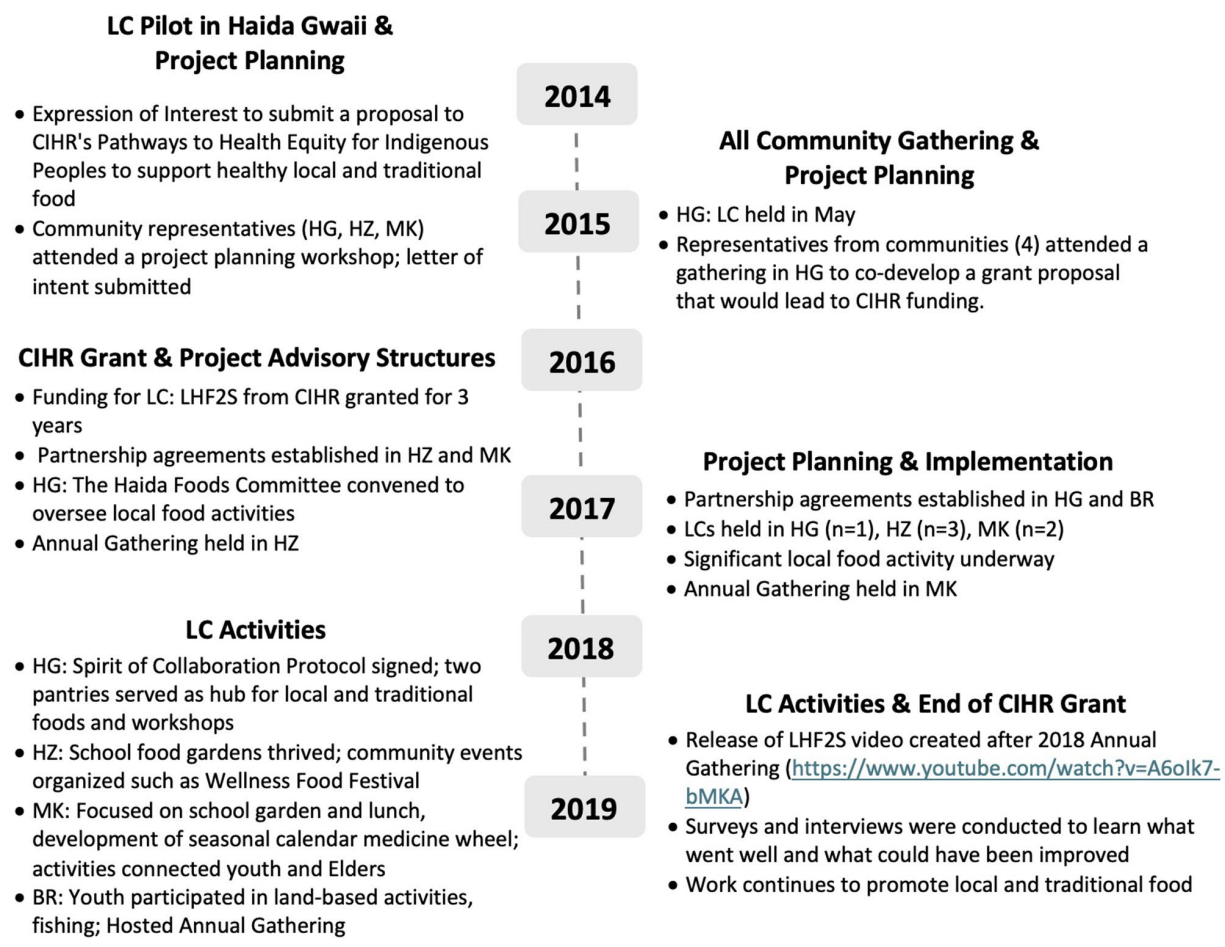
An overview of the Learning Circles: Local Healthy Food to School (LC: LHF2S) initiative and timeline. Community project activities are described over a 5-year period (2014-2019) from pilot work in Haida Gwaii and scale-up of the LC model within four partnering First Nations communities from 2016-2019: Haida Nation, Haida Gwaii (HG), British Columbia (BC); Gitxsan Nation, Hazelton /Upper Skeena (HZ), BC; Ministikwan Lake Cree Nation (MK), Saskatchewan; and Black River First Nation (BR), Manitoba

The four communities that chose to collaborate in the LC: LHF2S proposal had shared interest in strengthening their local and traditional food systems. At the 2014 and 2015 planning meetings, communities had the opportunity to learn about the work that was done in Haida Gwaii to implement the LC model and advance community food systems. Subsequent meetings throughout 2016 and 2018 took place in each of the communities (in-person and over teleconferences) to shape the work, enhance relationships and evaluate progress. Figure 1 provides an overview of the LC: LHF2S initiative timeline and community project activities.

### LHF2S: partnering communities and context

While communities were joined by a shared interest for local, healthy and traditional food, each community had distinct cultural, social, governance and geographic contexts. Details on the differing community contexts are described by McEachern and colleagues (2022) along with their varying goals and areas of activity [17, 19].

The people of Haida Nation embrace a strong relationship with the land and surrounding waters and are committed to strengthening relationships between the environment and wellness [15, 19, 31]. The traditional territory of Haida Nation is on the archipelago of Xaayda Gwaay.yaay (Haida Gwaii) with over 200 islands and encompasses parts of southern Alaska. Located approximately 100 km from the North Coast of British Columbia, Haida Gwaii is home to approximately 4,500 people, with most residing in two main areas, Gaaw Tlagee (Old Massett) at the north end of Graham Island and HlGaagilda (Skidegate) at the south end. The island is known for its vibrant traditional food culture and lengthy history of locally grown food. The island’s temperate climate provides a favourable season for many growers and gardeners to provide community members with access to a range of vegetables. Wild meats (e.g., venison), plant foods (e.g., berries, seaweed), fish (e.g., halibut, salmon) and shellfish (e.g., crabs, clams) are traditional foods commonly eaten. Initiatives to enhance access to local and traditional foods and food skills for school communities are a step towards improving food and nutrition security.

Engagement with Haida Gwaii began in 2014 with funding to pilot the LC model (previously referred to as Learning Labs). The work continued through co-development of subsequent funding and implementation of the current 2016–2019 phase, with the goal of enhancing Haida leadership in sustaining the establishment of two food pantries and distribution of food to local schools. The Secretariat of the Haida Nation and Haida Foods Committee comprising of Haida leaders and representatives of the school board provided strategic and administrative oversight of project activities.

The Gitxsan are encompassing of 14 distinct community-based populations within the Skeena watershed of north-western British Columbia [32]. Approximately 3,403 reside within the seven Gitxsan communities, while approximately 5,544 people make up the total population of all Gitxsan Wet’suwet’en communities and the two municipalities of Hazelton and New Hazelton. The landscape throughout the Upper Skeena area is mountainous, with surrounding vegetation of pine forests, hemlock, and cedar. Connection to land and embracing the sacredness associated with intimate knowledge and relationships from taking care of the land is of importance to Gitxsan [33]. Traditional food also remains of social and cultural importance to the community [18]. Salmon, wild meats, berries, and vegetables are commonly consumed traditional food. Prior to participating in the LC: LHF2S initiative, food insecurity and concerning rates of chronic diseases among youth were identified challenges to focus on through school-based food programming and on-the-land learning. Increasing access to local and traditional food, along with enhanced traditional food skills through expansion of gardens and greenhouses in schools were therefore identified as key goals through the LC [19]. The Gitxsan Government Commission (GGC), a tribal council, which provides administrative support to four local band councils (locally elected bodies that are administratively linked to the Government of Canada under the Indian Act), was engaged in collaboration with the Storytellers’ Foundation, an NGO chosen to provide administrative support for the project in this community.

Ministikwan Lake Cree Nation is a remote community within north-western Saskatchewan and has approximately 1,300 residents. Situated within a subarctic climate, cold weather generates a shorter growing season in the community. The community had a long history of gardening, however, through colonization, traditional food skills were lost as fewer government rations were provided to families that grew their own food [34]. Current high unemployment rates and limited availability of food, with the nearest grocery store being approximately 100 km away in the city of Meadow Lake, further constrain food access. Despite these challenges, food-land related activities remain of high importance to community members, including growing, fishing and access to hunting in the surrounding boreal forest. The goals of the LC therefore focused on school gardening, youth engagement, and traditional food skills. The LC initiative in this community was guided by a local LC advisory group comprised of Elders, a health director, community health worker, and community dietitian from Meadow Lake Tribal Council (MLTC). The MLTC comprises of representation from 9 local Meadow Lake First Nations, and is responsible for delivering a range of services to community members and the secretariat for the project [35].

Black River First Nation is an Ojibwa community on the banks of the O’Hanley and Black Rivers, and is situated along the east shore of Lake Winnipeg, Manitoba [36]. Situated within a humid continental climate and approximately 138 km northeast of the provincial capital city, Winnipeg, and 36 km north of Pinefalls, the First Nation has a population of approximately 1,500, with 1,000 people who live on-reserve [19]. The community is accessible year-round by a paved road, and the surrounding water and landscape are accessible via boat and snowmobile for harvesting and other traditional practices and activities. Harvesting of wild rice, hunting, commercial fishing, and agricultural development make up essential components of the community’s economic base. Prior to participating in the LC: LHF2S initiative, the community led a school garden and on-the-land learning program. Key goals of the work therefore focused on building traditional food skills and fostering relationships between Elders and youth through growing (e.g., fruit trees), fishing, hunting, harvesting of wild rice, and berry picking. Governed by a band Chief and Council (the Secretariat of the project), the community is also a member of the Southeast Resource Development Council Corporation which supports planning and delivery of community programs.

### ABLe Change Framework

Implementation science provides a suite of models, frameworks and theories to mobilize knowledge into practice [37, 38]. The ABLe (Above the Line, Below the Line) Change Framework (the Framework) is a strategic and conceptual tool to guide planning and implementation of community projects [28]. A guide to transformative system change, the Framework emphasizes a strong collaborative approach to defining the desired initiative, identifying key people to engage, and how they can co-plan change pursuits.

Based on a review of the literature on implementation science theories and models, the Framework was selected for its flexibility and emphasis on community engagement which closely aligned with participatory approaches. The Framework’s iterative and flexible process to working with partners is supportive of tailoring and adapting program planning and implementation to the priorities and needs of people and their local context. This includes a consideration for participatory engagement of those involved to support shifts and adaptions as needed [28]. Principles of community-based participatory research have supported decolonizing ways of working with Indigenous communities [39–44], and the Framework’s flexibility presents opportunities for linkages to other models that can inform community project plans. For example, the Framework’s emphasis on collaboration is particularly relevant to work involving Indigenous communities which requires relational-based approaches to information gathering such as large group conversational processes [45–47].

We therefore used the Framework to evaluate and describe the process of the LC in four diverse communities. Elements of the framework guided the evaluation of the LC: process, outcomes in terms of local food system activities, and factors that supported success within and across contexts.

**Fig. 2:**
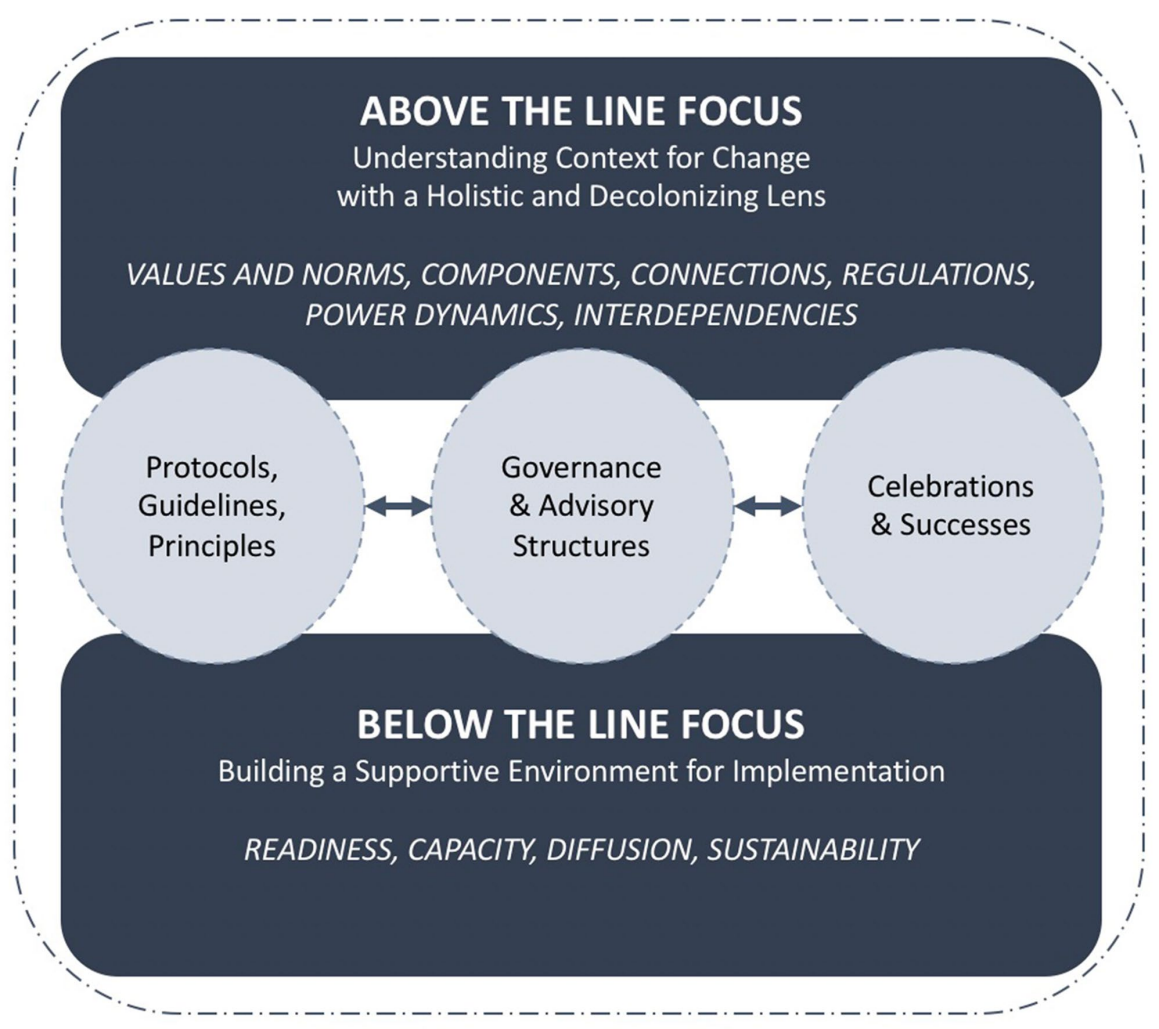
The ABLe Change Framework illustrating the *Above the Line* components for building an understanding for system change and the *Below the Line* components for enhancing state of readiness and capacity for change. Adapted and modified with permission from Foster-Fishman & Watson (2012) [25]

The main elements of the adapted Framework are described as Above and Below the Line (Fig. 2). Change pursuits are expected to simultaneously work within the Above and Below the Line components as they are interdependent processes that respond as actions are taken and partners are engaged. In adapting the Framework to this project’s context, modifications were made to better align and respond to implementation planning with Indigenous communities. Specifically, principles intended to link elements Above and Below the Line were modified to better integrate community knowledge and respectful approaches to working with community. Changes made to the Framework are described below.

The Above the Line component embeds systems change thinking to address the root source of the issue and to understand why the desired change is needed. It does so by engaging stakeholders to gather their perspectives on why the problem exists and the need for the desired change [28]. In the current project, we therefore, utilized the elements in the Above the Line component to focus attention to understanding context for change as aligned with local and traditional food system activities within school communities. This component guided considerations for how the LC facilitated space for dialogue among community members and stakeholders to understand and strategize ways to promote food security and food sovereignty. Within the Framework, we modified the Above the Line focus on “initiatives theory of change infused with a systemic lens” to “understanding context for change with a holistic and decolonizing lens” - a process regarded as essential to any work conducted with Indigenous communities [39–44].

Below the Line refers to the implementation process in supporting a climate (modified to ‘environment to support Indigenous values and connections to the land’) for food systems change and is focused on four key elements: readiness, capacity, diffusion and sustainability. This component guided consideration of features of the LC that support the implementation process. Both Above and Below the Line components are expected to work together to support an environment for transformative change [28]. We therefore modified and adapted strategies to better support relevance to Indigenous contexts. For example, systemic action learning teams identified as a strategy in the Framework, was modified to *governance and advisory structures* based on the current research and as informed by communities at the outset of project planning. Though ABLe Change does embrace a systemic lens which supports action research and whole systems considerations, its application within the Indigenous contexts of the current research required integration of decolonizing approaches, like community-based participatory research and holistic perceptions of health.

Other modifications made include simple rules (modified to *protocols*,* guidelines and principles*), and small wins (modified to *celebrations and successes* of milestones and short-term outcomes achieved). While small wins can be interpreted as having low impact, they are often seen as positive and meaningful changes within communities.

Modifications made to the Framework reflect approaches taken within the LC: LHF2S to honour Indigenous ways of working which included the following: applying Indigenous specific protocols such as the First Nations principles of ownership, control, access and possession [29], promoting participatory engagement, and utilizing Indigenous approaches such as storytelling [45–47]. Such approaches informed co-development of the initiative proposal and subsequent planning meetings with the project team and community partners. The decolonizing lens applied within the current study therefore supported a process for community engagement in shared decision-making regarding the research design, implementation, and evaluation of community food projects. The two advisory groups convened to govern the LC: LHF2S initiative and community LCF’s hired to oversee evaluation were essential to the community-led process undertaken to ensure activities and outputs were responsive to community priorities and needs.

### Data collection and sources

Community partners guided the research approach for data collection and analysis. Interviews (*n* = 52) were conducted by a member of the research team (LWM) and a trained community research assistant. Participants (*n* = 37) were purposely selected and recruited by email or in person. Each interview was approximately 25 and 60 min in length and took place by phone, Skype and in-person. Annual interviews (four sets in total conducted between 2015 and 2018) were conducted with LCFs, community members and other key partners using a semi-structured interview script. During the last annual gathering, in-person interviews took place following the final Annual Gathering. Annual Gatherings, which took place in each community, convened community members, researchers and project advisory members to build relationships, share project stories, engagement experiences, and evaluation activities. To protect participant anonymity, pseudonyms were assigned after data collection. All interviews were transcribed verbatim.

Data sources (Table 1) such as reports were developed by the LCF in each community which captured discussions, activities, action items, and planning steps with community members and food system actors. Other information captured in tracking sheets included timesheets, menu plans, food procurement data, workshops/food skills classes and communications emailed to the research team. These documents also included notes taken during Annual Gatherings, conference calls between project partners, and emails, which took place and were exchanged throughout the duration of the LHF2S initiative. Further details on the research process, including data collection, implementation and evaluation are available in other published materials by the co-authors [17–21].

**Table 1:** Data sources reviewed and thematically analyzed

Data Sources	Total Number of Sources/Responses
Activity tracking reports	39
Interviews with LC and Annual Gathering participants	52
Meeting summaries	44
Reports	11

### Thematic analysis

Data sources including interview transcripts, LC reports, meeting summaries, and tracking reports were thematically analyzed [48–50]. The data sources analyzed spanned the research planning, implementation and post-program evaluation phases (Table 1). As the ABLe Change Framework was used to guide analysis of scaling-up the LC, deductive reasoning was made based on the concepts and ideas from the ABLe Change Framework to code and interpret data. Data were thematically analyzed by the research team using a structured phased-approach as outlined by Braun and Clarke (2012) to synthesize findings across all communities [48–50]. Members of the team (BZ, LWM, RV) independently reviewed the data and generated initial codes, with the second coder (AD) conducting a deeper analysis to identify themes and synergies across the data initially coded by others. In addition, discussion with the project team of emergent codes and themes took place to ensure findings were accurate based on the standpoint and perspectives of the project leads and community partners involved. As such, intercoder agreement took place during the analysis phase, while discussions with the broader team supported consensus, validity and ensured alignment with community partner priorities. Further, the variety of data sources (Table 1) were triangulated [50] to build comprehensive themes reflective of community perspectives. Analyses provide a collective story of mediators for change, for example, key community champions for community-led action to take place. NVivo software version 12 Pro (QSR International) was used to code transcribed narratives.

## Results

Implementation of the LC approach in partnering communities enabled a participatory process for planning, capacity building, and action in local food systems. Across the four communities, successful implementation of the approach led to the following main outcomes: (1) development of a proactive community-driven response to food security and food sovereignty; (2) fostering of relationships and establishing trust between food system collaborators, key decision makers, and community champions; (3) advancement on the Truth and Reconciliation Commission call to action number 22 [51] through revitalization of traditional and local food systems to strengthen relationships between food, land, culture and health; and (4) enhanced community skills and dedicated resourcing through community food actions that support revitalization of local food systems.

The LC process evaluation results are described below according to key elements of the ABLe Change Framework (Table 2). Key themes and supporting quotations from community perspectives using the LC process to plan and facilitate local food system action are oriented by ABLe Change Framework components.

**Table 2:** Key themes to support the implementation of the LC model for collaborative and iterative planning-to-action in community food systems

ABLe Change Framework	Themes
Above the Line	
Components	• Engaging with community Knowledge Holders, local leaders and food system partners to understand community priorities
	• Identifying existing community strengths including models, programs, and services
Values and norms	• Incorporating local knowledge to understand community values and preferences for food system actions
Connections	• Promoting holistic wellness by restoring connections to land and culture through food
	• Acknowledging truth in tensions from colonization
	• Embracing Indigenous and Western worldviews through partnerships for food system planning and action
Power dynamics	• Establishing trust from the ground up to foster multi-level collaboration and commitment
Regulations	• Considering community governance, leadership and protocols
	• Working within federal, jurisdictional, and organizational level policies and regulations
Interdependencies	• Making strategic linkages between processes, programs and perspectives
Below the Line	
Readiness	• Supporting community readiness and food system partnerships
Capacity	• Strengthening capacity to effectively transform local food systems
Diffusion	• Meeting communities where they are at
Sustainability	• Taking action to secure funding stability
	• Partnering to maintain project activities and planning

### Above the line: who and what constitutes local and traditional food systems?

Key themes were identified within the six system characteristics outlined in the *Above the Line* component of the Framework. These include: (1) components; (2) values and norms; (3) connections; (4) power dynamics; (5) regulations; and (6) interdependencies. As such, themes highlight how the LC process supported understanding the characteristics of the food system and opportunities to strengthen local food access.

### Components of the system

The first element in the *Above the Line* planning phase of ABLe Change draws attention to understanding components of the system. Within LHF2S, understanding the range, quality and location of existing programs and supports for food system change was facilitated by community engagement under the LC process.

#### Engaging with community Knowledge Holders, local leaders and food system partners to understand community priorities

*“The Learning Circle offers a space where diverse views*,* passionate ideas and heated discussions are facilitated to develop a common vision and goals” [LCF 4*,* Annual Gathering participant]*.

As an initial step to inform action planning, community priorities were identified through engagement with Knowledge Holders who could collaborate to strengthen existing activities and address gaps identified.

*“So it’s trying to connect the people so that they can understand what each other is trying to do. And then trying to figure out a mechanism to make it possible – then removing barriers*,* especially to traditional foods. So yeah getting different perspectives to look at it*,* instead of looking at is just through*,* you know this is my system and this is your system” [LCF 1]*.

Within each community, the LCF worked with the community’s advisory committee to identify and collaborate with a range of people who had knowledge of the food system, school communities and wellness priorities. This collaborative work enabled LCF to leverage relationships with people committed to championing community food priorities and projects of interest.

*“I think the diversity of people that are at the circle*,* so we have elementary school teachers*,* we have high school teachers*,* there’s a number of folks from different governance levels in the community and also there’s farmers involved. I think that’s really critical*,* and there’s mainly women but a few men. And there’s a variety of ages too*,* right from some younger folk to old guys like me” [LC participant 3]*.

Through the LC process, communities were able to identify partners and community priorities related to programs and processes requiring improvement. For example, one community was focused on developing food pantries, a co-operative hub of frozen and preserved local and traditional foods, as one approach to improve local access to food. The pantries also served as a place where food activities were offered (e.g., canning workshops).

*“There are some strategic partnerships being built…pantries*,* LC*,* schools and farming community is engaged” [LC participant 4]*.

As Haida Gwaii initially piloted the LC model, scale-up in Haida Gwaii largely focused on enhancing Haida leadership through establishment of advisory structures such as the Haida Foods Committee. While ongoing activities were intended to enhance their food hub through the north and south food pantries, efforts were also focused on increasing engagement with Haida people to lead local food system activities. Development of a Spirit of Collaboration to guide the research process and relationship building was also informed through community engagements with Haida leadership. For the other communities, the initial emphasis focused on engaging key community members, food system actors, and other partners to collaborate in planning and build on identified strengths and community interests.

#### Identifying existing community strengths including models, programs, and services

Community engagement under the LC process provided an opportunity for LCF to identify and engage key people in the community who could shape actions for food system change and were invested in a culture of healthy, local, and, as emerged over the project, traditional food in school communities. As traditional food was valued by all communities, existing Knowledge Holders and programs that brought children on the land for traditional skills development were examples of existing strengths that were leveraged.

*“We are trying to go on a healing journey*,* emotional healing*,* spiritual healing*,* and physical healing. We are providing wilderness first aid; we have a medicine garden. We are going back to the ceremony. We have a caller*,* traditionally trained as a caller; the translation is come home – we are coming home to our knowledge systems*,* we are starting to train the teachers and the front-line workers. We have a healing garden and archery program” [Annual Gathering participant 1]*.

Priorities such as enhancing traditional teaching and skill building were addressed through initiatives such as overnight camps, medicinal plant teaching and river rafting for example.

*“There are two teachers at [school] who already do work with traditional and local foods in the school. The Cultural Teacher has been running a garden*,* smokehouse*,* medicinal plant walks and weeklong cultural hunting camps for over five years. Last year she secured funds to have a root cellar built at the school. There is interest from the Foods teacher to build a greenhouse at the high school to extend the season and have her students grow the food they cook with” [LCF 4]*.

Other existing program activities and supports identified through the LC process include a wellness model, school-based food programs, greenhouses, community gardens, and youth-based land programming. For example, in the Gitxsan Nation, the process of understanding community perceptions of wellness led to the development of a Wellness Model [52]. The Gitxsan within Hazleton/ Upper Skeena had collaborated to develop and disseminate a community informed wellness model and this served as a guiding lens within which the food system work could be situated.

*“The Gitxsan Wellness Model and the relationship of lax yip (of that land*,* Gitxsan knowledge) and otsin (spirit) to the work of connecting young people to their wellbeing through their relationships with culture*,* relations*,* food and land. The Wellness Model encompasses a holistic worldview in which the wilp (mother and relations) and the wilksawitx (father and relations) intersect and overlap with the lax yip and the otisin. All pieces are interconnected and wellbeing is expressed through the health and strength of these relations. Food cannot be separated out from all of the other relationships and is an integral part of Gitxsan wellbeing” [LC participant 6]*.

Integrating the wellness model into the LC meant promoting an awareness and understanding of traditional food as sacred and the process of bringing healthy traditional food to school to support holistic wellness.

### Values and norms

This component of the Framework draws attention to the attitudes, values and beliefs that may underlie perceptions of the problem and how desired changes can be addressed. Within LHF2S, the LC model facilitated the sharing of community perspectives and values related to local, traditional and healthy food. Efforts were centred on creating space for each community to share ways to improve access to and availability of local and traditional food.

#### Incorporating local knowledge to understand community values and preferences for food system actions

Communities highlighted the importance of connection to the land and intergenerational sharing of knowledge through the promotion of traditional food activities among youth. For example, development or expansion of school gardens were often explored to support skill building among youth and restore connections to culture and traditional food.

*“Shifting values around local food in the school culture would happen by building capacity to create more opportunities for children and youth to connect with food in a hands-on way” [L*C *participant 7]*.

As traditional food was widely valued by community members, activities that can promote traditional food knowledge and skills were emphasized. For example, developing a curriculum about traditional food and other local foods through in-person workshops and mentorship programs within schools were discussed in Haida Gwaii and Hazelton/Upper Skeena. Community Knowledge Keepers held workshops on traditional knowledge, for example about medicinal plants, and skills including food procurement (e.g., fishing) and preparation (e.g., filleting and drying).

*“The teachings for preparing food traditionally*,* that are based on values*,* sustainability*,* and survival*,* are important to share” [Annual Gathering participant 2]*.

Building capacity to engage in traditional food activities and growing of local food was viewed as a pathway to reconnect youth with the culture, language and traditions of the community.

*“Teaching respect for the food and the places that it comes from*,* you know it’s really helping to bring people together around the traditions and the cultural practices around food” [LCF 1]*.

### Connections

Emphasized in the Framework is the importance of relationships and making connections with key people who can inform planning and processes to embark upon changes of interest. Building connections across systems and sectors, like educators and food producers, can help to improve coordination between services and strengthen partnerships. It can also support the building of trust amongst local organizations and ways to work together to share resources, information or partner on an initiative.

The LC model was perceived as an approach that facilitated connections among people, programs, and organizations. It allowed for efforts to be coordinated to maximize benefits to the community; identification of areas where trust needed to be established; opportunities to build partnerships; and engagement that helped heal relationships. In addition, the approach enabled communities to make connections between different multi-sector partners at community, regional and jurisdictional levels.

*“The Learning Circle brought partners together who may not have otherwise engaged around food. It was exciting to have the Gitanmaax Nursery School attend the last Learning Circle as their participation is helping to bridge the project from pre-school age to school age. Many of the Nursery school staff are Gitxsan*,* are active gardeners*,* and knowledgeable about food systems” [Annual Gathering participant 3]*.

#### Promoting holistic wellness by restoring connections to land and culture through food

*“To connect with our spirit-being on the territory*,* eating traditional foods all help to take care of the spirit” [Annual Gathering participant 2]*.

Emphasized was the importance of revitalizing connections between food and culture as a pathway to holistic wellness. Among youth in particular, promoting teachings in school about traditional food and land-based activities was encouraged. Engaging the community in more traditional food activities and restoring connections to land and culture by bringing healthier food to the community was identified as a priority.

*“So learning how to enjoy foods in a way that’s accessible I think is part of it. How do you make healthy food attractive and delicious and I think that’s a skill set that* *many people have lost. And so even in the learning circle there was a cool opportunity to share some of that knowledge back and forth. Where it was like ideas about “oh this is how you can get kids to eat this” [LC participant 9].*

#### Acknowledging truth in tensions from colonization

While support for locally grown and traditional food among youth and school communities was viewed as an important avenue for restoring connections between food, health, culture and land across all communities, many emphasized the importance of recognizing and understanding the impact of colonization within this context. In particular, recognizing tensions between farming and agriculture associated with the loss of traditional land and environmental contaminants from pesticides was discussed with intensity by one community. This was different than the experiences in the other three communities, which emphasizes the diverse influences colonization has on each community.

*“Commenting on the “farm to school” concept*,* many children were sent to residential school and had to work in gardens/farms. The notion of a farm to school idea brings negative connotations to it. It is important to learn as much as you can*,* be sensitive*,* and consider history” [Annual Gathering participant 3]*.

Remaining sensitive to how gardening and farming may not always have a positive association was emphasized even when the intent and outcome was related to community priorities for healthier food.

*“When it comes to farming*,* I think there for sure is that tension of farming was not typical. Although there was tending and stewardship*,* farming is not a traditional practice” [LC participant 11]*.

#### Embracing Indigenous and western worldviews through partnerships for food system planning and action

*“Recognize that self-determination is an important or central goal of reconciliation between First Nations and Canadian government. We need to work together to demonstrate that projects need to be centred around Indigenous ways of knowing and adequately support First Nations health and well-being” [Annual Gathering participant 7]*.

Under the LC process, space was created for community members to draw on the strengths of Indigenous knowledge and ways of life, and perspectives of non-Indigenous partners engaged in project planning. In doing so, community members were able to build relationships, share ways of knowing and doing, and identify opportunities for collective actions. LC engagements offered opportunities to make connections with other members of the community and their work which supported coordination of activities to improve access to traditional and locally farmed food, and promote culturally appropriate ways of growing, harvesting in schools and beyond. For example, donations received from farmers and fishers at food pantries were able to provide food to schools and hospitals.

Other LC activities focused on relationship building, gardening, developing food skills, and connecting youth to traditional food activities and the land. Building relationships with community members and establishing partnerships allowed for the opportunity to identify ways to partner. In Hazelton/Upper Skeena, for example, the building of relationships with farmers, schools and key organizations such as Gitksan Health and First Nations Health Authority supported integration of local procured foods into school programs and fostered engagement at community-wide organized events.

*“These are small communities and it’s hard to know what relationships result in ongoing connection*,* although I would say it was for sure a positive experience*,* the Learning Circle*,* for people who were part of it and they felt connected to each other” [LCF 5]*.

LC participants felt that connections were able to be made between projects focused on food to support actions in the community. In addition, Annual Gatherings created space for cross-community learnings and relationship building to support actions within individual communities.

### Power dynamics

Within the Framework, this component highlights the importance of understanding how decisions are made and identifying who participates. This consideration is of particular importance to any work with Indigenous communities. Adopting a decolonizing approach such as participatory processes can be a way to redress power imbalances by centring Indigenous voices in decision making and promoting community empowerment.

#### Establishing trust from the ground up to foster multi-level collaboration and commitment

All participating communities emphasized the importance of meaningful engagement to build relationships and establish trust. Trust and commitment to community were consistently viewed as not only essential to ensure accountability of those working with community, but for reconciliation and a path forward to heal from the impacts of racism and colonization. Having honest conversations about the impacts of colonization and intergenerational trauma was acknowledged as important for building trust and relationships. These conversations were important within each community and at the Annual Gatherings across communities to develop trust among partners, researchers, LCF and other community members. The conversations helped to develop a shared understanding of how colonization and residential schools affected individuals and communities. Space for sharing openly with each other and consider how that might influence the action taken by each community. Community advisors identified, for example, the need to acknowledge trauma associated with colonial land appropriation for agriculture and forced farm labour of students attending Indian Residential Schools before proceeding with gardening activities within one of the communities.

*“Healing can’t start until the wounding is finished…the time is now to heal the wounds from colonization. We all live here; we all need to work together” [Annual Gathering participant 4]*.

A first step towards healing wounds was engaging key leaders with decision making power and trusted members of the community involved with implementing on-the-ground activities. However, as one participant pointed out, fear and a lack of openness to reflect and learn about the history of colonization are barriers for non-Indigenous people to embark on a path towards reconciliation with Indigenous peoples.

*“There’s a fear piece about how to talk about current effects of colonialism without sending non-Indigenous people into this fear reactive*,* defensive place when it’s maybe something somebody hasn’t talked about. And from other work [community organization] is doing from around the work of internal reflection and dialogue that I feel non-Indigenous people need to do…I really feel like*,* with reconciliation*,* there is work that both Indigenous and non-Indigenous people need to do” [LC participant 12]*.

Within the research team, Annual Gatherings became an opportunity for engagement of non-Indigenous team members with Indigenous advisors and community members. They were an opportunity to build relationships and respect for diverse knowledge and strengths. They also afforded insight into the unique cultures of participating communities who took turns in hosting the four gatherings. The openness to learning by the research team and flexibility, for example, in letting communities determine how success for the project should be defined and evaluated, helped build trust. In turn, the community advisors were generous in supporting research team interests, like academic theses or papers, that did not directly benefit the community.

*“The hope for the project is that university*,* partners*,* Learning Circle facilitators and communities treat this as a true partnership in which the strengths that we each bring to the project are valued and that challenges can be faced openly” [LC participant 4]*.

Within Haida Gwaii for example, the initial ‘learning lab’ school food work was established without substantive Haida leadership. As such, a goal of vertical scale was to enhance Haida leadership and community engagement to support and sustain ongoing efforts. Haida leadership was integrated through the Secretariat of the Haida Nation to manage funds from the project and provide administrative support. In addition, convening a project advisory committee, *X*aayda (Haida) Foods Committee (HFC), was a way to ensure decisions made throughout the project were by and for community. This advisory committee was established in 2016 to guide project activities and facilitate partnerships. A memorandum of understanding called the “Spirit of Collaboration” (Isda ad dii gii isda (S)- Isdaa ‘sgyaan diiga isdii (M)) was co-developed and signed between the HFC and the University of Waterloo in 2017 to guide evaluation efforts. Such an agreement helped to align the research scope with community priorities and cultural values and promote the self-determination of programs implemented.

*“Over time*,* we have been able to develop partnerships and processes*,* and especially [the Haida Foods Committee]*,* which is now able to – in addition to the Learning Circle*,* provide me [a non-Indigenous person] with guidance on how to move forward appropriately with food programming in our communities. And how to properly work towards following and communicating of protocols – [Indigenous community] protocols” [LCF 1]*.

For the LC in Upper Skeena, partnerships were established between the Storytellers’ Foundation, a non-governmental organization based in the community since 1994, and members of the Gitxsan Government Commission (GGC) tribal council that provided direction and advisory support for the project [17]. A partnership agreement was established in 2016 between the University of Waterloo and Storytellers’ Foundation that enabled the organization and community advisors to lead the hiring of the LCF and manage local project related costs. This process can be viewed as an approach that prioritizes community involvement in decision-making to address tensions that may arise from differential power relationships. Meetings held as part of the LC approach helped to initiate a process of engagement and shared understanding of priorities and goals for the community. Storytellers’ held the funding agreement and prioritized the work to be led and informed by community on activities focused on food, active living, healthy eating, food sovereignty and land-based activities. Moreover, the community had pre-existing partnerships with several organizations involved with food security initiatives. Additional partnerships, include Skeena Watershed Conservation Coalition, Hazelton Secondary School, Majagaleehl Gali Aks School (MGA) and Senden Agricultural Resource Centre, that participated in shared decision-making processes to understand perspectives of local, healthy, and traditional foods in schools within the community through the LC [17].

*“I think there’s huge community support for this type of work in the Hazelton region and the Upper Skeena region. The agricultural community and the farming community got onside as much as they could and members of that community like farmers and growers like [individual; organization] have been involved in more of an ongoing leadership process” [LCF 5]*.

Community action planning within Ministikwan Lake Cree Nation involved engagement with Meadow Lake Tribal Council and an established advisory committee with Indigenous leadership. Leadership from such partners supported greater engagement from community members in planning efforts around traditional food activities and bringing healthy food to community. The LC model provided the opportunity to address power dynamics that arise when someone outside of the community is engaged in the work. Trust and relationship building to bring members from community along the LC journey was recognized as a process requiring time and meaningful engagement. In addition, community members shared that tensions and divides within the Indigenous community itself arise when uncoordinated efforts among leadership are left unaddressed.

In Black River, power was centred within an Indigenous context from the start. Black River First Nation identified a number of key partners to engage in planning efforts including the Band Council as a way to ensure support was led and informed by community. Key partners included Nanaandawewigamig First Nations Health and Social Secretariat of Manitoba, Food Matters Manitoba and HSF.

### Regulations

Policies and practices that influence system behaviour are acknowledged within the ABLe Change Framework as key factors to consider when thinking about action and system change. Key areas considered under this component include any organizational policies that may be a barrier or enabler for action and how existing policies or procedures may support achievement of the overall goal. Within the LHF2S initiative, consideration for community-level protocols and working within jurisdictional food policies were an important step in planning under the LC process.

#### Considering community governance, leadership and protocols

*“Right to land*,* right to harvest*,* protocol*,* how does that work*,* like one of the circles I remember asking the question- you know teachers were talking about going out and picking soap berries… so then I posed the question*,* who do you ask to go? How do [you] get out there? Who do you have to ask and what do you have to put into place to take your class and go do that?” [LC participant 12]*.

Understanding the importance of working within local community governance, the project team including the LCF relied on Elders for direction on relevant decision making. For example, working with the Chief and Band Council System and/or Tribal Council/ Secretariat for support was viewed as essential by community advisors, recognizing their influential role in community-level decision-making of practices, policies and programs implemented. As such, each community’s governing system was engaged in planning efforts, including administration of funds and decisions for how resources would be used for local food action. In addition, formalized advisory committees established informed non-Indigenous partner organizations of specific community protocols for appropriate engagement to be followed.

Working within an Indigenous governing system and establishing a community (Indigenous)-led committee was recognized as a way to seek guidance for evaluation efforts and ensure that it respected local Indigenous ways.

*“I think Haida Foods Committee is something I’m really*,* really happy about because I feel that I can actually now do my job with much more comfort than before….in terms of connecting with other partners and sharing information*,* and just being able to access additional resources” [LCF 1]*.

#### Working within federal, jurisdictional, and organizational level policies and regulations

While First Nations communities in Canada are governed by their own leadership (elected, e.g., Band Council or ancestral e.g., hereditary Chiefs) they also fall under regulatory governance at Canadian federal and provincial levels. Recognizing Canada’s multi-level jurisdictional environment, which brings differences in policies and practices on food security, food safety, and land use practices, was important.

*“In terms of the national Indigenous health strategy*,* there is a lot of policy work at the national level*,* nation to nation; at the community level they are looking at providing training to students*,* have heart smart program that has Indigenous health incorporated into it*,* talks about some Indigenous health concepts” [LCF 3]*.

While some policies and strategic plans developed may support and accelerate actions and priorities identified through LC engagements, others considered some health and safety regulations as a challenge to promoting uptake of traditional foods and integration into schools.

*“There is the structure of health and safety regulations for food in Canada. Basically*,* make Indigenous traditional food that is harvested*,* prepared and served to*,* throughout the Indigenous community and non-Indigenous communities here*,* basically says that is no good*,* it is not good enough by the health and safety regulations that exist” [LC participant 11]*.

### Interdependencies

#### Making strategic linkages between processes, programs and perspectives

Recognizing how each of the Above the Line elements interact and reinforce each other is highlighted in the Framework. Focused efforts on understanding the values of community members, for example, may inform other areas to support planning such as characteristics of the food system and programs that communities would like to see more aligned with community preferences.

*“It’s taking lots of players and bringing them together. And then they all have their own networks and it’s a really good way to make connections in the food world*,* or any kind of thing that you’re working on. But it gets people out of their silos and gives an opportunity to work towards common goals” [LCF 1]*.

As the LC are fluid, their processes can be shaped by priorities identified and membership changes over time. Identifying and engaging new people within the community can help with identifying shared interest, resourcing, and opportunities for new relationships that can strengthen efforts to plan, overcome challenges and sustain project activities.

### Below the line: building a supportive environment for implementation

The Below the Line element of ABLe Change is focused on aspects to support successful implementation and ensure that planning efforts achieve intended outcomes. Elements of the Above the Line focus on understanding system characteristics, also help to inform Below the Line components which consist of *readiness*,* capacity*,* diffusion*,* and sustainability*. The LC model applied by each community enabled a process to assess and build on existing strengths, levels of readiness and capacity for food system action. In addition, efforts to raise awareness about the initiative (diffusion) and planning to maintain actions brought about by bringing more healthy food to school (sustainability) were made by community members at LC meetings.

### Readiness

#### Supporting community readiness and food system partnerships

Readiness has been described as a state in which those involved who will be impacted by change efforts understand and believe that action is necessary, feasible and desirable [25]. At LC meetings, the various people convened were better able to understand challenges and opportunities for greater food sovereignty, and brainstorm ways to integrate local and traditional food among youth.

Awareness of the proposed change, its feasibility and desirability among those leading change efforts is an important part of successful implementation. As community engagement is essential to planning that reflects the preferences and priorities of the people directly impacted by the change, LC meetings and cross-community annual project gatherings allowed for readiness to be advanced at various levels including organizational, leadership, and community, by identifying where strengths and gaps exists. Community gatherings enabled a process for understanding local context, food experiences, interests and reflect on how the LC model could evolve in each community.

Organizations such as University of Waterloo, Farm to Cafeteria Canada, the Heart and Stroke Foundation, and Native Women’s Association Canada were key partners supporting cross-community vision and project activities. Partners were engaged in building a shared understanding and awareness of community food systems considerations such as the influence of external environments like market food systems or climate change.

*“One thing definitely I think there are a lot more food security initiatives*,* a lot more awareness on food security*,* there is awareness that living in the Northwest we are at risk of you know*,* we’ve been seeing lots of things happening around us- droughts*,* fires*,* all of those kinds of things. So*,* we really need to think about having our food here” [LCF 5]*.

Fostering awareness of food system actions is also important to building strong partnerships with community members and leadership who can shape and drive change efforts. Communities shared the importance of obtaining support from local leaders and champions such as the school principals, teachers, committees, Chief and Band Council or Tribal Council. Champions were identified as playing a strong influential role in decision-making, planning engagements with a broad range of food system actors within school communities and supporting integration of local food into schools.

*“It’s the fact – that person has to be invested in wanting to buy local…and having it become policy that they source local first” [LC participant 14]*.

*“It will be important to mobilize people. There are other meetings and gatherings in between the LC where work can happen. People need to commit to working together. Bringing together people with different perspectives and priorities around food” [L*C participant 4*]*.

Engaging local leadership helped to ensure broad community participation toward shared awareness, trust, and a sense of ownership of activities. Such an approach has helped to generate excitement and enthusiasm by community members to be engaged in action planning and lead on-the-ground change efforts.

*“Connecting with other partners and sharing information*,* and – and just being able to access additional resources. Whether they’re financial or otherwise*,* I feel like we can – we can do that much better collectively. And with strong leadership from them*,* because then there’s – there’s a great deal more trust” [LCF 1]*.

Understanding and working through interdependencies between elements outlined in the ‘Above the Line’ component helped to enhance the state of readiness for food system actions. For example, understanding components of the system such as existing programs and new initiatives helped with facilitating connections, cross-cutting priorities and building of relationships. Further, the trust and respect established through built relationships and formal partnerships, can support navigation through power dynamics and policies that may either function as a barrier or facilitator for food system actions.

### Capacity

#### Strengthening capacity to effectively transform local food systems

Building community capacity (e.g., skills and knowledge sets) [28] was at the forefront of LC planning efforts. The LCF played an essential role in strengthening capacity by leading engagement activities and fostering connections with key food system actors to collaboratively champion food priorities and projects of interest. Community-based meetings and cross-community annual project gatherings provided the opportunity to share learnings and leverage successful approaches taken to support planning and implementation in and across respective communities.

*“Share teachings so we are able to learn together and be aware. –create space where we can stand in our own truth*,* be who we are*,* and be safe there. –openness-non-judgmental atmosphere in order to allow us to learn. –we have to show up with our baskets full (what is my own culture*,* where do I come from*,* what is my truth? engaging as people from a place of truth). –we are always teachers*,* students*,* and learners. –respectful dialogue among nations-there is always more to learn” [Annual Gathering participant 8]*.

With support by the LCF to organize a workshop, communities were able to engage in a process to identify a vision and key actions to support community-identified priorities. For example, in Hazelton, their goals consisted of four areas: build relationships, build gardens, learn about food, and share information. One of the objectives to support this overarching vision of the community was to have a garden and greenhouse in each school that would broaden students’ knowledge, experience and skills with local food through curriculum-based projects. As such, actions were taken with two elementary school gardens and a partnership with Skeena Watershed Conservation Coalition (SWCC) for students from New Hazelton Elementary to grow food and host a seed-planting workshop with the students. In addition, with a grant from Farm to Cafeteria Canada, students from Majagaleehl Gali Aks Elementary School were able to start a garden and local food salad bar in the school.

*“Most people felt that shifting value around local food in the school culture would happen by building capacity to create more opportunities for children and youth to connect with food in a hands-on way” [L*C *participant 21]*.

Contingent capacity is inclusive of system knowledge, relationships built between organizations who can support management of the change, and system actors to ensure availability of resources to support with change efforts [25]. Leadership provided through commitment from local governance helped to obtain funding support to promote change capability and support for project activities to take place in the community.

### Diffusion

#### Meeting communities where they are at

Diffusion is described as a purposeful spread, adoption and use of the change of interest [28]. The LC was often identified as an approach that is flexible and can be adapted to best meet the needs of where communities are at in their level of readiness and capacity to begin planning for food system actions.

*“The model [LC] presents as an accelerated way of building on a set of capacities to move a school towards deeper procurement of local food for the students*,* but that assumes that there are farmers or hunters and gatherers that have a regular yield at a sufficient level and to the safety standards that a school could buy. It also assumes that the school would have the storage facilities and a local champion and coordination abilities and requisite food standard protocols all established in order to facilitate that. So the model is good in the sense that it’s flexible and can be adapted to different levels of community capacities for this kind of change….but there is no mechanism presently in place for assessing the level of capacity of the willing participating schools and related partners” [LC participant* 18*]*.

At annual cross-community project gatherings people shared the benefits of the LC and interest in its continued use as an approach to bring various perspectives together, facilitate identification of synergies in priorities and opportunities to work together. There were other forms of diffusion including word of mouth and various communications related to the project within a community.

*“[Our] project is not only having a positive impact on the participating community*,* but the effects are spreading out*,* especially with* [Tribal Council] *being connected to 9 communities*,* circles are extending” [LCF 3]*.

LC workshops and Annual Gatherings that brought partnering communities together enabled a process to support awareness of each community’s project objectives and activities and ways to modify and improve the application of LC in each community. In Haida Gwaii where a LC goal was supporting more Indigenous leadership and engagement, it became clear that some of the processes of the LC [e.g., planning them on a teacher’s professional development day (Pro-D day)] were not working for the First Nations schools that were part of a different school jurisdiction. While planning a LC to engage teachers in public schools worked on a Pro-D day, it was not a good way of engaging teachers from First Nations schools. Efforts were made for greater engagement of First Nations schools at the subsequent LC.

*“You can adapt a learning circle to have more Indigenous people if it’s not working. We didn’t get tons of Indigenous people at this Learning Circle because it had to be on a Pro-D day. Can we go to the [First Nation] school and get your involvement? What works for you*,* how could we get your involvement?” [L*C*F 5]*.

Annual Gatherings permitted diffusion across contexts as participants from each program shared their perceptions of what success looked like, what processes were working well and experiences or outcomes people were proudest of. The group also brainstormed about responses to challenges faced. Through this process, participants brought new ideas back to their community.

### Sustainability

Sustainability is an important element of action planning and implementation, ensuring that project activities or outcomes are maintained through policies or practices. Communities were encouraged to think about sustainability in their engagements and at each planning meeting.

Sustainability was an ongoing focus topic of discussion within the context of each project to identify ways to continue work across each community. Though each community worked within their local context and different stages of readiness and capacity, stable funding and diverse partnerships were identified as key sustainability strategies.

#### Taking action to secure funding stability

The LC: LHF2S project provided research funding support for a LCF in each community to assist with the LC process and project evaluation. Implementation and infrastructure support were not provided by the research grant. For example, funding for school food programs, was sourced from within communities and external sources, as shared among communities. It is noteworthy that in the exemplar context of Haida Gwaii, the established project had identified sustained funding sources within and external to community. This enabled, for example, two pantries to be staffed and operate as local food hubs where stored locally sourced traditional foods could be accessed for a fee by schools or other community programs. Other communities had varying levels of internal and external funding support for priority initiatives. Considerations for long-term and consistent funding to support resources required to manage and maintain project activities beyond CIHR funding were therefore made by communities.

*“For a local food to school food system to be sustainable there must be funds in place to start and keep programs going otherwise it will just be another pilot project that fizzles out in a few years. Additionally*,* teams and relationships must be built so that it’s not just reliant on one or two champions that may move on without the momentum in place to keep the programs going. We talked about sustainability in terms of generations*,* how the experiential learning children do now can shift value around food to further generations” [LC participant 21]*.

*“Having that ability to sustain yourself is hugely important*,* so it’s already kind of happening here and people are just kind of “oh great” so now we can use this little bit of funding to be able to support our kids to learn this in more of a formal way” [LCF 5]*.

*“Once the funding for the Learning Circle evaluator facilitator is not here in this community anymore  we will see whether those structures can stay. We’re really finding that sort of external support*,* the schools are definitely telling us that its needed*,* that it helps support with different grants and that sort of thing” [LC participant 11]*.

The importance of ongoing and additional funding support was a fundamental priority expressed by all communities as key for sustaining LC engagements and building economic support for food security projects.

#### Partnering to maintain project activities and planning

Other key considerations made from cross-community discussions on project sustainability included the importance of partnerships that can support sharing project successes with other communities; creation of a plan to continue cross-collaboration among the communities to foster knowledge exchange and opportunities to align efforts to obtain funding. Building strong relationships was recognized as necessary to identify connections between programs, continue the momentum established from projects, and identify supports required to manage project activities.

*“Connecting with other partners and sharing information*,* and – and just being able to access additional resources. Whether they’re financial or otherwise*,* I feel like we can – we can do that much better collectively. And with strong leadership from them*,* because then there’s – there’s a great deal more trust” [LCF 1]*.

The importance of relationships and key partnerships in supporting ongoing work and maintaining actions beyond a funding cycle was highlighted. Reflections were shared at LC meetings on ways to improve project planning and setting up communities for success, such as having tools and resources to build capacity for implementing food system actions. One suggestion was a pre-project assessment that can serve as a planning tool to better understand levels of readiness and capacity that would position communities to move ahead with their projects of interest. Such a tool could be used to facilitate relationship building and partnerships to plan for sustainability.

*“So in my view what it needed is a pre-initiative capacity assessment that would chronicle in detail the different levels of capacity or rather the different levels of capacity attainment across different domains of capacity….You’ve got an assessment*,* you place yourself and there’s some menu of options of this version of the model that would best fit your particular circumstances and then you work through the mechanics of how that would work” [LC participant 18]*.

### Celebrations and successes (“quick wins”)

Even though different communities were at different levels of readiness and capacity, they all managed to implement activities related to healthy local or traditional foods. So, while one community may face challenges with limited resources or capacity for multi-stakeholder engagements through LC workshops, they did, over the time of the project, experience small wins in facilitating school-based food programs on the land.

## Discussion

We used the ABLe Change Framework to examine how the LC model as scaled-up across four communities facilitated capacity building for community-led actions to strengthen local and traditional food access and skills in partnering First Nations school communities. A process led by communities, the LC approach facilitated collaboration with multi-level partners to encourage community actions to enhance local and traditional food availability in school communities. The LC enabled a participatory process for community members and partner organizations to collaborate in understanding community priorities, context for project development, and strategies to create a supportive environment for local action.

Collectively, the LC facilitated a process for communities to: (1) build on strengths and explore ways to increase readiness and capacity to reclaim traditional and local food systems; (2) strengthen connections to land, traditional knowledge and ways of life; (3) foster community-level action and multi-sector collaboration and relationships; (4) drive actions towards decolonization through revitalization of traditional foods; (5) improve availability of and appreciation for local healthy and traditional foods in school communities; and (6) promote holistic wellness through steps towards food sovereignty and food security.

Implementation science scholars have often pointed to the importance of understanding context for change and for the process to be informed by the people such changes are intended to serve [5, 28, 53, 54]. Participatory approaches that can facilitate a collaborative process are therefore fundamental to program development that is responsive to community priorities. As documented in this paper, LC can offer such an approach to facilitate co-planning for collaborative and community-driven action.

LC supported a community-engaged, iterative and dynamic process for understanding *why* healthy, local and traditional food are needed; *who* should be engaged, and *how* key decision makers, implementers and champions can work together within the community. In addition, the LC allowed for an understanding of key components that make up local food systems, as well as identification of where strengths and gaps may exist. By collaborating with a broad range of food system actors and leaders, communities were able to create a vision for their food system that builds on community-identified strengths, traditions and resources.

As LC’s are intended to bring various people together across and within each community, creation of a safe space was a fundamental component of the approach that centres Indigenous voices and addresses power dynamics that may arise from different perspectives. This process prioritized Indigenous perspectives and supported an interactive process for communities to engage in participatory decision-making on local food actions.

Being community-driven, the LC approach was also adaptable to diverse community contexts. Though partnering communities were joined by their shared interests in strengthening local food systems, each community worked within its context, level of readiness and capacity for local action. Flexibility built into the LC approach allowed for each community to modify and adapt its process based on community-specific needs. For example, in Haida Gwaii, vertical scale-up of the LC helped to sustain structures, like pantries, and growth in actions to support food system planning. Horizontal scale-up across the three new diverse contexts was also able to support other activities ranging from the expansion of community gardens and greenhouses in Hazelton/Upper Skeena to building a single garden in Ministikwan Lake, and planting of fruit trees in Black River.

While some communities had supportive relationships established with community leadership and organizations that accelerated planning, others leveraged the LC to support efforts on building awareness and relational capacity by identifying key people within the local food system to engage (e.g., food producers, consumers, community Knowledge Holders, community-based organizations and public health). For example, Haida Gwaii extended capacity to transition leadership to the Council of Haida Nation and the Haida Foods Committee of the LC and evaluation activities. This was a key process that helped centre Indigenous peoples voices and reclamation of traditional food systems within the work. A memorandum of understanding for research also embedded principles for non-Indigenous researchers and community members to work together in a good way. The Haida Foods Committee also continues to serve the community’s research needs with respect to ethics and ownership moving forward.

The horizontal scale up across the three new diverse contexts has shown that even over a short time period, the LC concepts of bringing people together to plan, resulted in some positive action to enhance local and traditional food access and skills for youth. Elements like relationships formed and early successes may support sustainability. For example, in Hazelton/Upper Skeena, the LCF supported the building of relationships with farmers, schools and key organizations such as First Nations Health Authority. Through such connections, local procured foods from farmers were integrated into school food programs [17, 18]. Within Ministikwan Lake Cree Nation, local champions, a community health worker and school cook, were important for supporting a school lunch program and promoting healthy eating in the community. While champions proved to be key enablers for program planning, relying on a small number of people in the community also came with challenges [17–21]. In particular, one community champion expressed concerns regarding burnout from multiple requests and competing priorities. Smaller communities, as was the case with Black River in the current project, may not have had the range of food system actors to realize the benefits of the LC model experienced in larger communities. Limited capacity in Black River presented challenges with hiring a community member as the LCF. Nevertheless, collaboration amongst those with a shared interest did facilitate some positive actions, such as school trips on the land.

With the LC extended in diverse contexts, the documented successes of the LC presented opportunities for continued use and adoption in other First Nations communities. As food security and food sovereignty remain a priority for First Nations communities in Canada, calls have been made for initiatives that can support communities with building capacity, as well as maintaining or enhancing access to and availability of traditional food [55]. This includes efforts to establish culturally appropriate school-based programs that can ensure access to healthy foods [55]. Based on the cross-context analysis guided by the ABLe Change Framework, the LC model can be adapted to different Indigenous contexts. In particular, in communities where there is interest in community food actions, the use of LC can be explored to facilitate discussion, engagement with a range of local food actors and commitment on priority plans.

To support successful implementation of LC in other communities, this research suggests that the following elements are key: (1) a LCF who has knowledge of the community and established relationships; (2) local champions to support activities, whether the LCF or others; (3) support of local leadership and advisors; and (4) strong partnerships.

In community discussions about farming, the importance of acknowledging impacts of colonization, intergenerational trauma and racism was emphasized as part of the process for reclamation of local food traditions. A lack of awareness of history and racism within school communities may therefore function as a barrier to building trust and stronger relationships with schools as well as integration of traditional food and cultural teachings. Creating space within LC meetings to acknowledge the ongoing impacts of colonization on local food systems can support promotion of reflexivity and awareness among non-Indigenous LC participants as a way to bridge stronger relationships and embrace Indigenous knowledge and worldviews in action planning. Such considerations of settler-colonial relations and how they have disrupted Indigenous food systems including connections to the land has been emphasized as important for addressing food security [1, 3, 7, 8].

This participatory project with First Nations communities responds to the broader call made by scholars which emphasize the importance of decolonizing approaches to Indigenous health research [42, 43, 56, 57]. While LC played an important role in facilitating actions towards decolonization through revitalization of traditional foods and promotion of holistic wellness, there is a need for Indigenous-led models or frameworks that can better support planning and implementation. There are opportunities for growth within this field. In planning the current study, we scanned the implementation science literature and found that a limitation of all available frameworks was that they did not provide guidance for sustainable implementation of health promoting and equity-focused programs and services intended to serve communities [54], let alone Indigenous communities.

Use of ABLe Change at a grassroots or community-level would require reconceptualization to better align with community-specific principles and values. As an implementation framework, the concept and elements offered by ABLe Change could also be made more accessible by creating an interactive tool to work through a critical assessment within the Above and Below the Line elements of the Framework. This would better support users of the Framework with development of an action plan for implementation. In utilizing the framework to guide the current analysis, attention was drawn to opportunities for growth within the field of implementation science to better meet the needs of Indigenous communities. In particular, the importance of relationships as foundational to supporting collaboration throughout all phases of work. This is consistent with the call for decolonizing approaches to community research and knowledge mobilization [5, 54, 58–61].

ABLe Change can incorporate participatory action research and its inherent flexibility, relational approach and potential for sustainability are strengths of the framework. However, it falls short in centring Indigenous self-determination and equity. An Indigenous specific tool that can support action within participatory-based research and Indigenous principles would offer a great contribution to implementation science scholarship and practice. The findings shared from this evaluation highlight the LC model as a participatory approach with communities, but recognize the need for new methodologies to draw from decolonizing research and an Indigenous paradigm. Such attention to decolonizing approaches of inquiry, has also been highlighted by scholars who emphasize the importance of research with Indigenous communities to be centred on self-determination, engagement and collaboration to navigate power dynamics and prioritize community voices [11].

## Conclusions

The LC is a community-based participatory approach that fosters multi-sector and community collaboration. LC supports a community-led process to build on existing strengths to plan and implement community-identified priorities and activities for enhancing access to local, healthy and traditional foods in school communities. Insights from a participatory implementation initiative, LC: LHF2S, were drawn from an evaluation of LC as a participatory approach to facilitate capacity building and action planning in school community food systems within four partnering diverse First Nations communities across Canada.

An initiative co-developed and implemented with communities, the LC supported collaboration within and across each community and formal partnerships between Indigenous and non-Indigenous organizations to engage in an iterative and interactive planning process for community-led action. Utilizing an implementation framework, ABLe Change, as a guide to assess how LC functioned to enable critical elements of effective implementation, we highlight how LC supports a participatory process that allows for communities to identify *what* changes are needed, *who* should be engaged in planning and decision making, and *how* information is gathered and shared. Further, flexibility built within the LC approach enabled scale-up across diverse community contexts and presents opportunities to further scale-up the approach to accelerate steps towards food security and food sovereignty elsewhere. While implementation science offers a range of supporting resources to ensure program planning efforts are set up for success, impact and sustainability, the field calls for greater integration of decolonizing methodologies that can facilitate participatory community-driven work but also promote self-determination and health equity in Indigenous communities.

## Data Availability

The data that support the findings of this study are available but restrictions apply to the availability of these data, which were used under license for the current study, and so are not publicly available. Data are however available from the authors upon reasonable request and with permission from community partners of the project (Haida Nation, Gitxsan First Nations, Ministikwan Lake Cree Nation and Black River First Nation).

## Abbreviations

ABLe: Above the Line, Below the Line
BC: British Columbia
HFC: Xaayda (Haida) Foods Committee
LC: Learning Circles
LCF: Learning Circles Facilitator
LC:LHF2S: Learning Circles: Local Healthy Food to School
MB: Manitoba
MLTC: Meadow Lake Tribal Council
OCAP: Ownership, Control, Access and Possession
SK: Saskatchewan
GGC: Gitxsan Government Commission
